# The promise of human embryonic stem cells in aging-associated diseases

**DOI:** 10.18632/aging.100328

**Published:** 2011-05-11

**Authors:** Odessa Yabut, Harold S. Bernstein

**Affiliations:** ^1^ Cardiovascular Research Institute, San Francisco, CA 94143 USA; ^2^ Eli and Edythe Broad Center of Regeneration Medicine and Stem Cell Research, San Francisco, CA 94143 USA; ^3^ Department of Pediatrics, University of California San Francisco, San Francisco, CA 94143 USA

## Abstract

Aging-associated diseases are often caused by progressive loss or dysfunction of cells that ultimately affect the overall function of tissues and organs. Successful treatment of these diseases could benefit from cell-based therapy that would regenerate lost cells or otherwise restore tissue function. Human embryonic stem cells (hESCs) promise to be an important therapeutic candidate in treating aging-associated diseases due to their unique capacity for self-renewal and pluripotency. To date, there are numerous hESC lines that have been developed and characterized. We will discuss how hESC lines are derived, their molecular and cellular properties, and how their ability to differentiate into all three embryonic germ layers is determined. We will also outline the methods currently employed to direct their differentiation into populations of tissue-specific, functional cells. Finally, we will highlight the general challenges that must be overcome and the strategies being developed to generate highly-purified hESC-derived cell populations that can safely be used for clinical applications.

## I. Aging-Associated Diseases and Stem Cell Therapy

In 2010, individuals aged 65 years and older constituted approximately 12.9% and 8% of the population in the United States and worldwide, respectively [1]. This number is expected to increase dramatically as millions of individuals from the baby boom generation born between 1945 and 1964, continue to reach this age. Thus, the ability to prevent and treat aging-associated diseases is rapidly becoming a primary focus in various sectors of the biomedical field.

Aging-associated diseases include degenerative conditions affecting tissue and organ function. For example, neurodegenerative disorders such as Alzheimer's disease, Parkinson's disease, and amyotrophic lateral sclerosis (ALS) are conditions marked by the progressive deterioration of structure and function leading to neuronal death. A retinal disorder, age-related macular degeneration, is caused by the gradual degeneration of cells in the macula of the retina and is the leading cause of vision loss in adults over age 55. Conditions such as osteoarthritis and osteoporosis, which are marked by the degeneration of cartilage and bone, respectively, cause the majority of knee, joint, hip, and spine injuries in older individuals.

Aging-associated diseases may also arise from cell dysfunction. Such conditions may include cancer, heart disease, chronic obstructive pulmonary disease (COPD), and diabetes. Cancer is caused by metabolic changes in cells that lead to DNA damage that can fuel the uncontrollable and inappropriate proliferation of cells. The risk of cancer increases significantly with age. Heart disease is typically caused by prolonged exposure of the heart to hypertension, hypercholesterolemia,diabetes, and other cardiovascular risk factors, as well as an age-dependent increase in the prevalence of left ventricular hypertrophy, diastolic dysfunction, and atrial fibrillation [2]. COPD is a group of progressive diseases of the respiratory system that includes emphysema, characterized by the destruction of alveolar cells lining the lung epithelia, and chronic bronchitis, which is caused by abnormal mucus production along the bronchial airways [3]. In the case of ‘adult-onset’ type 2 Diabetes, pancreatic islet β-cell function can be impaired such that insufficient insulin is produced, or cells become resistant to insulin [4].

**Figure 1.**
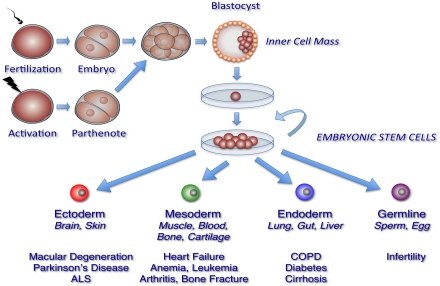
Generation of pluripotent human embryonic stem cell lines. Generation of human embryonic stem cell (hESC) lines involves several steps. Donor embryos are first obtained after *in vitro* fertilization or by egg activation (parthenogenetic embryos), and allowed to develop *in vitro*. Pluripotent cells are then isolated either from the inner cell mass of pre-implantation blastocysts or from 4, 8, or 16 -cell stage morulae. Finally, isolated cells are plated in defined hESC medium with or without feeder cell layers to propagate and select for pluripotent cell populations. These processes have resulted in hESC lines able to generate tissues from all three embryonic germ layers and the germline.

The prospect of repairing or replacing damaged, dysfunctional or missing cells with new functional cells has shifted the therapeutic paradigm toward restoring tissue function in individuals affected with aging-associated diseases. The primary candidate for the development of these therapies is stem cells, particularly human embryonic stem cells (hESC), which has the capacity to self-renew indefinitely and differentiate into all tissue-specific cell types (Figure 1). In this review, we will describe the derivation, maintenance, and properties of pluripotent hESCs. We will also outline the methods used to induce the generation of specific cell types from hESCs, with primary focus on cell types that are applicable in understanding the pathology, as well as a potential source of cell-based therapies, in aging-associated diseases.

## II. Human Embryonic Stem Cells: Sources, Maintenance, and Common Properties

Human embryonic stem cells (hESCs) are pluripotent stem cells derived from various stages of embryonic development. hESCs are uniquely capable of proliferating indefinitely and differentiating into all tissue cell types. The unrestricted potential of hESCs has made these cells especially attractive for therapeutic applications. In particular, the regenerative capacity of hESCs could be the key to successful treatment of aging-associated diseases which, as discussed in the previous section, are characteristically marked by progressive dysfunction and/or loss of somatic cells.

**Table 1.** Methods for differentiating hESCs into specific cell types for treatment of aging-associated diseases

	Clinical Application	Cell Type	Method	Specific Factors and/or Conditions	Ref.
**Derivation of Endodermal Cells from hESCs**	Cirrhosis, Hepatocellular carcinoma, Diabetes-associated liver disease	Hepatocytes	Differentiation of hESC into definitive endoderm, followed by sequential exposure to differentiation factors	FGF, BMP4 hepatocyte growth factor oncostatin M dexamethasone	[35, 36]
	Diabetes	Pancreatic Islet Progenitors		Activin A, Wnt3A keratinocyte growth factor/FGF7 retinoic acid cyclopamine Noggin	[33]
	Chronic obstructive pulmonary disease	Lung Alveolar Cells	Genetic modification of hESCs followed by spontaneous differentiation	Recombinant keratinocyte growth factor	[38, 39]
**Derivation of Mesodermal Cells from hESCs**	Prevention and treatment of infection, allograft rejection, allergic and autoimmune diseases, and targeting cancer cells	Dendritic cells	Human embryoid body formation	Serum-free conditions BMP4	[102]
		Blood cells	Spin embryoid body formation	Serum-free conditions	[41]
		T and NK cells	Co-culture with stromal cells	Co-culture with stromal M210-B4 cells to enhance expansion of CD34^+^/CD45^+^ progenitors	[43]
	Degenerative joint and bone diseases	Chondrocytes	Human embryoid body formation	Micromass of dissociated embryoid bodies BMP2	[54]
				High density culture of dissociated embryoid bodies Ascorbic acid dexamethasone	[57]
			Directed differentiation on 3D scaffolds	Co-culture with primary chondrocytes poly-D, L-lactide scaffold	[56]
	Heart disease	Cardiomyocytes	Human embryoid body formation	Serum-free conditions bFGF	[46]
			Directed differentiation	Activin A BMP4	[51]
				BMP4 BMP4/bFGF/Activin A VEGF/DKK1 VEGF/DKK1/bFGF	[52]
			Genetic modification	Cardiac-specific reporters	[49, 53]
**Derivation of Ectodermal Cells from hESCs**	Parkinson's disease	Dopaminergic neurons	Co-culture with stromal cells	FGF8 Shh	[61]
			Formation of neural rosettes	FGF8 Shh	[66]
	Alzheimer's disease, Huntington's disease	Cholinergic neurons	Formation of neurospheres	Shh, FGF8, BMP9 *or* LHX8/GBX9 overexpression	[68]
	ALS	Motor neurons	Formation of neural rosettes	Retinoic acid Shh	[67]
		Schwann Cells	Formation of neural rosettes	ciliary neutrotrophic factor neuregulin 1β dbcAMP	[64]
		Oligodendrocytes	Directed differentiation	B27, thyroid hormone retinoic acid, FGF2, EGF, insulin	[65]
	Age-related macular degeneration	Retinal pigment epithelium		Serum-free conditions Activin A, nicotinamide	[73]

### A. Sources and Derivation of hESCs

hESCs are typically derived through the microsurgical removal of the inner cells mass (ICM) of the blastocyst-stage pre-implantation embryo (Figure 1). Cells populating the ICM are pluripotent, in that they are capable of differentiating into the extraembryonic endoderm and the three germ layers that form all tissues of the embryo: ectoderm, mesoderm, and endoderm. Under specific conditions, these cells can proliferate in the undifferentiated state *in vitro* and retain their pluripotency indefinitely. hESCs have also been derived and established from single blastomeres of the 4- or 8- cell embryo [5-8], 16-cell morula [9, 10], or the ICM of parthenogenetic embryos. A single blastomere is highly totipotent and can generate an entire embryo. Thus, hESCs derived from blastomeres circumvent the ethical controversies surrounding the use of hESCs, since the removal of a single blastomere, in theory, will not impede the ability of the remaining blastomeres to form a normal embryo. Similarly, parthenogenetic embryos, which are generated through artificial fertilization of donor oocytes [11-14], have become highly desirable sources of hESCs because viable embryos are neither created nor destroyed. Furthermore, hESCs derived from parthenotes are especially attractive because these cells are homozygous for major human lymphocyte antigen (HLA) alleles, which could help circumvent immunological rejection that may occur in hESC transplantation therapies (discussed below). Since the initial derivation of pluripotent hESCs from ICM by Thomson and colleagues in 1998 [15], hundreds of hESC lines have been established from various embryonic sources and are now utilized in basic and clinical research worldwide. In the United States, there are over 80 hESC lines that adhere to federal guidelines [16], some of which are now being used in clinical trials.

### B. Cellular and Molecular Properties of Pluripotent hESCs

**Figure 2.**
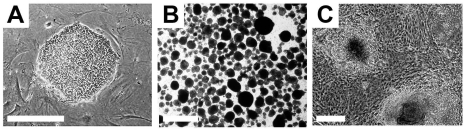
Typical undifferentiated and differentiating hESCs in culture. (**A**) A compact colony of proliferating pluripotent hESCs can be seen when cultured in defined medium on mouse embryonic fibroblasts. (**B**) Floating hEBs observed at 3 days after induction of differentiation. (**C**) Differentiating tissues, including cardiomyocytes, appear within adherent cultures at 48 hours after plating hEBs onto a gelatin-coated culture dish. Bar, 25 μm.

Pluripotent hESCs maintain specific morphological and molecular properties that are shared by the majority of hESC lines. Morphologically, single hESCs have an enlarged nucleus and distinct nucleoli. In culture, proliferating hESCs form compact cell colonies of spherical cells (Figure 2). Under differentiation conditions, these colonies lose their compact morphology and are distinguished by the appearance of flattened cells at the edges as differentiating cells begin to migrate out of the colony. Spontaneous differentiation of hESCs in culture can be controlled with regular supplementation of fresh growth medium [17].

A panel of molecular markers has been identified in 59 independently derived pluripotent hESC lines by the International Stem Cell Initiative, a consortium of stem cell researchers from more than 15 countries [18]. These markers have been routinely used when characterizing pluripotent hESCs. These include genes with known roles in maintaining pluripotency or other developmental processes such as Nanog, POU domain class 5 homeobox 1 protein (POU5F/OCT4), teratocarcinoma-derived growth factor 1 (TDGF1), DNA (cytosine-5-)-methyltransferase 3β (DNMT3β), γ-aminobutyric acid (GABA) A receptor β3 (GABRB3), and growth differentiation factor 3 (GDF4). Additionally, pluripotent hESCs express a number of surface markers such as Stage Specific Embryonic antigens 3 and 4 (SSEA-3, SSEA-4), along with keratin sulfates (TRA-1-60, TRA-1-81, GDTM2, and GCT343) and protein antigens (CD9 and Thy1). Furthermore, hESCs can also be identified based on the expression of alkaline phosphatase, stem cell factor (SCF/c-Kit ligand), and class 1 HLA proteins. Efforts are ongoing to profile the expression of microRNAs in pluripotent hESCs. Although a comprehensive study of miRNA expression is still lacking, several studies have identified a number of microRNA clusters prominently expressed in hESC lines, some of which have established roles in maintaining the pluripotent state of hESCs [19, 20]. These include microRNA (miR)-92b, miR-302 cluster, miR-200c, miR-368, and miR-154* clusters, miR-371, miR372, miR-373*, miR-373, and the miR-515 cluster [21, 22]. Pluripotent hESCs also display distinct epigenetic properties. Generally, the chromatin structure of hESCs is in an open conformation that allows transcription factors to enter and regulate gene expression [23]. In addition, DNA methylation profiles of hESCs are distinguishable from other cell types. Markedly reduced methylation patterns of CpG dinucleotides are specifically present in the promoter regions of pluripotency genes such as OCT4 and Nanog [24]. These unique epigenetic properties of hESCs are necessary to maintain their pluripotent state, and can therefore be used as a hallmark of undifferentiated hESCs.

### C. Establishing Pluripotency of hESCs

**Figure 3.**
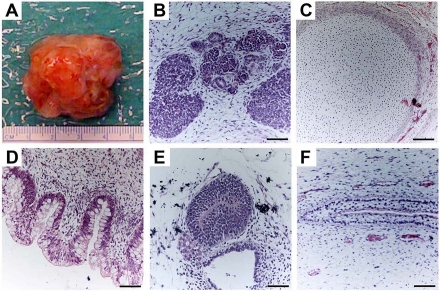
Teratoma formation provides an *in vivo* assay of hESCs differentiation capacity. Proliferating cultures of hESCs were used to form teratomas by renal capsule grafting using established methods [25-28]. (**A**) An explanted teratoma is shown. (**B-F**) Teratomas were sectioned and stained with hematoxylin and eosin to identify embryonic tissues. Representative tissues from all three embryonic germ layers can be seen, including mesoderm (**B,C**), endoderm (**D**) and ectoderm (**E,F**). (**B**) Nascent renal tubules and glomeruli within bed of primitive renal epithelium. (**C**) Cartilage surrounded by capsule of condensed mesenchyme. (**D**) Glandular intestinal structure. (**E**) Nascent neural tube. (**F**) Primitive squamous epithelium. Bar, 100 μm.

hESCs are capable of differentiating into cells that constitute the three germ layers. This can be tested using established *in vivo* and *in vitro* techniques that are routinely used to determine pluripotency of a hESC line. The most commonly used *in vivo* method involves the induction of teratoma formation after transplantation of undifferentiated hESCs into immunodeficient mice [25-28]. Teratomas are benign tumors consisting of tissue structures derived from the three embryonic germ layers (Figure 3). Analysis of teratomas formed from engrafted hESCs can be used to determine their differentiation potential. The ability of hESCs to differentiate into various cell types can also be tested *in vitro* through the formation of embryoid bodies (EBs). EBs are spherical colonies of non-adherent, differentiating hESCs that contain cell populations representative of all three embryonic germ layers. Under suitable conditions, EBs can differentiate into specific cell types. As will be discussed below, EB formation is typically used as an intermediate step when directing hESCs into tissue-specific cell populations.

## III. Differentiation of hESCs Into Specific Cell Types

The most promising feature of hESCs is the ability to derive lineage-restricted progenitors that are capable of differentiating into specialized post-mitotic cell types that can be used in cell-based therapies. Furthermore, hESCs provide a virtually inexhaustible source of specific cell populations, due to their ability to divide indefinitely. Current research studies are focused on identifying and refining ways for directing the differentiation of hESCs to enrich for pure, homogenous populations of specific cell types that can be used to either replace damaged cells or coax neighboring cells to function properly. In the following sections, we will provide some examples of how differentiation of hESCs is directed towards tissue-specific cells, particularly those with potential to treat aging-associated diseases.

### A. hESC-Derived Endodermal Cells

Endodermal derivatives include cells that populate the lung, liver, pancreas, urinary bladder, pharynx, thyroid, parathyroid, and digestive system. The initial step in generating endodermal cells is the formation of definitive endoderm. D'Amour et al. [29]showed that selective induction of definitive endoderm can be achieved through the addition of high concentrations of Activin A, under low serum conditions, and in a stage-specific manner. Activin A mimics the action of Nodal, a ligand that activates transforming growth factor-β (TGFβ) signaling. The effect of Activin A in inducing definitive endoderm is enhanced when additional factors are present, such as Wnt3a [30], Noggin [31], or when coupled with the suppression of the phosphoinositide 3-kinase pathway [32].

The induction of definitive endoderm from hESCs can lead to the generation of specific progenitor populations such as pancreatic islet β cells, hepatocytes, or alveolar epithelial cells. These are being developed with the intent of treating diseases such as diabetes, liver disease, or lung disease, respectively. Among the most successful examples to date is the generation of pancreatic islet progenitors devised by Kroon et al. [33] through the sequential exposure of hESCs to Activin A and Wnt3A, followed by the addition of keratinocyte growth factor or FGF7 to induce the formation of the primitive gut tube. Subsequently, retinoic acid, cyclopamine, and Noggin are added to inhibit Sonic Hedgehog (Shh) and TGFβ signaling, and thus induce the differentiation of posterior foregut cells, the source of pancreatic cell progenitors. These are cultured further to generate pancreatic endoderm cells. When these cells were engrafted into immunodeficient mice, they displayed the histological and structural characteristics of pancreatic islet β cells and were able to sustain insulin production for at least 100 days. These results have been met with great enthusiasm for their potential in treating diabetes, type 2 diabetes being the most common type affecting more than a quarter of individuals aged 65 years or older in the United States [34].

In a similar manner, hepatocytes are obtained after differentiation of hESCs into definitive endoderm [35, 36]. A highly robust population of functional hepatocytes was generated with the sequential addition of low serum media, collagen I matrix, and hepatic differentiation factors that include FGF, BMP4, hepatocyte growth factor, oncostatin M, and dexamethasone [36]. These cells expressed known markers of mature hepatic cells, exhibited appropriate function, and were able to integrate and differentiate into mature liver cells when injected into mice with liver injury. The ability to differentiate hepatic cells could prove useful in the treatment of a number of liver diseases that are prevalent in aging individuals, such as cirrhosis, hepatocellular carcinoma, and diabetes-associated chronic liver disease [37].

The use of hESCs to treat lung injury has also been an area of active investigation. A significant step towards directed differentiation of lung-specific cells was reported by Wang et al. [38, 39], in which genetically modified hESCs carrying lung-specific reporters under the control of promoters from tissue-specific genes such as surfactant protein C, aquaporin 5 and T1α, resulted in purification of type I and type II alveolar epithelial cells. When engrafted into mice suffering from acute lung injury, these cells exhibited functional properties including the capacity for gas exchange and histological amelioration of lung injury.

### B. hESC-Derived Mesodermal Cells

Directing the differentiation of hESCs into mesoderm requires the activation of the TGFβ signaling pathway and can be accomplished through the stepwise and dosage-dependent addition of Activin A, BMP4, and growth factors, such as vascular endothelial growth factor (VEGF) and basic FGF (bFGF) [40]. Mesodermal derivatives have also been successfully obtained by spontaneous differentiation of hESCs through hEB formation without first directing them toward mesoderm. Robust differentiation of hESCs into hematopoietic lineage cells, which give rise to all blood cell types and components of the immune system, has been achieved under serum-free conditions through spin hEB formation [41]. Specific hematopoietic lineage cell types, such as functional dendritic cells, have been successfully differentiated from hESCs through spontaneous hEB formation under serum-free conditions with the addition of BMP4 at specific time points [42]. Hematopoietic progenitor cells that give rise to functional T and natural killer cells capable of targeting human tumor cells both *in vitro* and *in vivo*, have also been derived from hESCs co-cultured with stromal cells [43]. Thus, the ability to differentiate hESCs into hematopoietic lineage cells promises to be useful in improving existing therapies that require blood cell transplantation, in fighting cancer, and in immune therapies that require induction of the immune response in an antigen-specific manner [44].

Cardiomyocytes, which represent another therapeutically important derivative of mesoderm, have been successfully generated from hESCs using several methods [45]. Through hEB formation, hESCs can spontaneously differentiate into cardiomyocytes under appropriate culture conditions. These cardiomyocytes exhibit morphological, molecular, and electrophysiological properties similar to adult cardiomyocytes [46], and display quantifiable responses to physiological stimuli reminiscent of atrial, ventricular, and pacemaker/conduction tissue [47-50]. Cardiomyocytes have also been generated by directed differentiation with Activin A and BMP4 on a dense monolayer of hESCs; these cells successfully form functional cardiomyocytes when transplanted *in vivo* [51]. Another study used additional medium supplements that included VEGF, and the Wnt inhibitor, Dickkopf homolog (DKK1), followed by the addition of bFGF, to promote cardiomyocyte differentiation from hEBs [52]. Success of these studies was measured by the expression of proteins specific for mature cardiac cells such as cardiac troponin T, atrial myosin light chain 2, and the cardiac transcription factors, Tbx5 and Tbx20. Several groups have generated cardiac-specific reporter hESC lines that can be used to test various differentiation strategies [49, 53] and generate specific cardiomyocyte subtypes [49].

hESCs can readily form connective tissue, such as bone or cartilage, as can be appreciated from teratoma formation assays (Figure 3). Thus, hESCs may be a valuable source of cells suitable for connective tissue replacement therapy for diseases such as osteoarthritis and osteoporosis, which are characterized by the breakdown of cartilage in joints and pathological fractures due to low bone density, respectively. Most successful and efficient protocols for directing chondrocyte differentiation from hESCs utilize 3D culture systems created by seeding hESCs at high density leading to formation of a pellet, or by introducing the cells into a synthetic 3D scaffold. Such systems enable cell-cell signaling between the undifferentiated hESCs and mature chondrocytes to stimulate homogeneous and sustained chondrogenic differentiation. For example, single-cell suspension of dissociated hEBs cultured as high-density micromass with BMP2 facilitates chondrocyte formation [54]. hESCs co-cultured with primary chondrocytes or in the presence of osteogenic supplements and polymeric scaffolds yield cartilaginous- or osteogenic-like cells [55, 56]. More recently, feeder-free 3D culture systems have successfully derived multipotent connective tissue progenitors from hESCs yielding tendon-like structures. The engraftment of these *in vitro* differentiated tendon structures in injured immunosuppressed mice restored ankle joint movements that rely on an intact Achilles tendon [57]. Furthermore, there is evidence to suggest that cell transplantation promotes growth and repair through endogenous cells as well [58].

### C. Ectodermal Derivatives of hESCs

The dominant differentiation pathway in hESC cultures leads to the formation of ectoderm, which gives rise to cells of the nervous system and the epidermis. hESC-derived neural progenitor cells are characterized by rosette-like neural structures that form in the presence of FGF2 or EGF through either spontaneous differentiation from an overgrowth of hESCs, or after hEBs are plated onto adherent substrate [59, 60]. These ‘neural rosettes’ have become the signature of hESC-derived neural progenitors capable of differentiating into a broad range of neural cells in response to appropriate developmental signals. Thus, many studies are exploring ways to enhance the formation of neural rosettes to generate enriched populations of specific neural cell types. One example is the use of stromal cell lines [61], which provides ectodermal signaling factors required for neural induction, and promotes the formation of neural rosettes [62, 63].

The withdrawal of FGF2 and EGF, and addition of other factors can lead to the differentiation of neural rosettes into specific neural subtypes. Neural crest stem cells derived from neural rosettes can differentiate into peripheral sympathetic and sensory neurons through the addition of BDNF, GDNF, NGF and dbcAMP, or into Schwann cells in the presence of CNTF, neuregulin 1β and dbcAMP [64]. Neuroglial cells, such as oligodendrocytes, are generated with B27, thyroid hormone, retinoic acid, FGF2, epidermal growth factor, and insulin [65]. Additionally, FGF8 and Shh induce hESC-derived neural progenitors to differentiate into dopaminergic neurons [66], while treatment with Shh and retinoic acid induce motor neuron differentiation [67]. Recently, functional basal forebrain cholinergic neurons have been derived from hESCs through the formation of neurospheres and subsequent exposure to Shh, FGF8, and BMP9, or by overexpression of LHX8 and GBX9 [68].

The ability to differentiate hESCs into neuronal and non-neuronal subtypes has generated much interest due to their potential use in drug testing or cell replacement therapies for a number of neurodegenerative diseases. In particular, the successful derivation of dopaminergic neurons, particularly those of the midbrain subtype, could potentially be used to treat Parkinson's disease, which is marked by the progressive loss and dysfunction of these neurons. In Alzheimer's disease, where the degeneration of basal forebrain cholinergic neurons causes debilitating cognitive dysfunction, hESC-derived cholinergic neurons may also be useful for therapy. However, hESCs may also be helpful without requiring cell replacement. As observed in a clinical trial in which autologous fibroblasts programmed to express human NGF were implanted in the forebrain of individuals with mild Alzheimer's disease, a marked improvement in the rate of cognitive decline was observed [69]. One can imagine exploiting genetically-modified hESC-derived neuronal progenitors that readily engraft and express therapeutic gene products, such as NGF, to prevent the degeneration of cholinergic neurons in the basal forebrain.

Similarly, hESC-derived motor neurons might be used in the treatment of ALS, which is characterized by the progressive loss of motor neurons in the cortex, brain stem, and the spinal cord. Studies of ALS disease models have also suggested that non-neuronal cells, such as oligodendrocytes and Schwann cells, may be involved in the pathogenesis of this disease [70, 71]. Thus, the ability to differentiate hESCs into both neuronal and non-neuronal cells of the central nervous system provides an attractive therapeutic approach.

The efficacy of transplanting hESC-derived oligodendrocytes to treat acute spinal cord injury is now being tested in the first clinical trial with hESCs to be approved by the United States Food and Drug Administration (FDA) [72]. Oligodendrocytes are rapidly lost during acute spinal cord injury leading to demyelination and neuronal loss. In a trial sponsored by Geron Corporation, purified oligodendrocyte progenitor cells derived from hESCs will be injected into the spinal cord of paralyzed patients within two weeks after injury. While this first trial is a safety study, the expectation is that these progenitor cells will terminally differentiate into oligodendrocytes and produce myelin, which insulates neuronal cell membranes and is critical for efficient conduction of neuronal impulse transmission. If successful integration and function of oligodendrocytes is achieved in these studies, it could lead the way toward new treatment approaches for ALS, which manifests in demyelination of degenerating motor neurons.

Retinal pigment epithelium (RPE) cells are another specific cell type derived from neuroectoderm. RPE cells support the neural retina by phagocytosing and renewing the photoreceptor outer segments of rhodopsin. Recent reports have shown that RPE can be induced from hESCs in the presence of nicotinamide and Activin A under serum-free conditions [73]. hESC-derived pigmented cells exhibit the morphological and functional properties of RPE cells after transplantation in an animal model of macular degeneration, a disease caused by dysfunction and loss of RPE. These data have led to the second and third FDA-approved clinical trials using hESCs, these sponsored by Advanced Cell Technology. For these trials, hESC-derived RPEs will be transplanted directly into the degenerating retinae of patients with Stargardt's Macular Dystrophy, a juvenile form of macular degeneration, or Dry Age-Related Macular Degeneration, to rescue visual acuity. The launch of these three clinical trials heralds the translation of hESC research into therapy for degenerative disease, and the fields of stem cell biology and geriatric medicine await the results with great anticipation.

## IV. Current Challenges and Potential Solutions for the Therapeutic Use of hESC-Derived Cells

Cellular therapies involving hESCs are in development and have begun to enter clinical trials. The International Stem Cell Banking Initiative (ISCBI) has been created by the International Stem Cell Forum, a group of national and international stem cell research funding bodies, to develop a set of best practices and principles for banking, testing, and distributing hESCs for therapy [74]. In the United States, the FDA also monitors these guidelines and have issued recommendations for reviewers of proposals for stem cell therapeutic trials [75]. It is important to note that these recommendations do not ensure the quality or efficacy of hESC-derived cells used for clinical applications. Rather, these guidelines warrant that the cells used for therapy are reproducible and meet specific criteria to ensure patient safety (Table 2). The major safety concerns for the use of hESCs are discussed in the following sections.

**Table 2.** Standardization and Quality Control of hESCs for Clinical Use

Requirement	Methods of Testing
Cell line identity	Short Tandem Repeat (STR) testing Human Leukocyte Antigen (HLA) testing
Sterility and pathogens	Bacteria/fungi/mycoplasma culture qPCR analysis for murine viral short interspersed elements (SINE)
Genetic/chromosomal stability	Single Nucleotide Polymorphism (SNP) analysis G-band karyotype analysis spreads Fluorescent in situ hybridization
Epigenetic stability	MicroRNA profiling Methylation analysis X-inactivation
Pluripotency	Teratoma formation SSEA-3/4, TRA-1-60, TRA-1-81 detection
Quality and differentiation ability	Gene expression profiling qPCR analysis Embryoid body formation
Functional assays	Potency Efficacy Lot-to-lot variability

### A. Xenobiotic-Free Conditions

Many of the hESC lines currently in use have been exposed to animal products during isolation and propagation of hESCs *in vitro*. Under these conditions, hESCs could possess animal viruses and other unknown substances capable of eliciting a detrimental immune response in transplanted hosts. Currently, hESC lines under development for clinical use undergo extensive microbiological testing as strictly recommended by ISCBI. In the United States, the FDA legally requires documentation of the source, the potential genetically modified components, and pathogenic agents in any hESC-derived cell intended for therapeutic use. Thus, avoiding exposure to xenobiotics is an ongoing effort. Recently, replacement media have been developed that would allow maintenance of hESCs in xenobiotic-free conditions. These include xenobiotic-free serum replacements such as Knockout Serum Replacer (Invitrogen) or xenobiotic-free culture media such as HESGRO (Millipore) or TeSR (STEMCELL).

Feeder-free culture systems are now being developed to reduce the risk of contamination with foreign agents when hESCs are cultured on feeder cell layers. Feeder-free and xenobiotic-free defined culture media that consist of a combination of recombinant growth factors known to inhibit differentiation and maintain hESCs in the pluripotent state are now commercially available. However, some reports have associated feeder-free culture conditions with greater chromosomal instability and an increased risk of propagating genetically altered hESCs [76]. For this reason, most hESC labs practice a surveillance program for genomic instability in cultured lines [28, 49].

hESC lines derived using human feeder cells have also been reported. For example, hESC lines have been successfully derived on human fibroblasts generated from neonatal foreskin [77, 78] and adult skin fibroblasts [79]. Some laboratories deriving new lines have moved exclusively to xenobiotic-free conditions [80]. The ability to derive and maintain new hESC lines using human fibroblast feeder cells represents a significant step towards generating clinical-grade hESCs.

### B. Genetic Abnormalities In hESC Lines

The best characterized hESC lines to date are among the earliest lines derived. However, they may not be the best lines for therapeutic application as many of these lines were derived using animal products. Chromosomal and genomic instability has been detected in several hESC lines, with acquisition of loss of heterozygosity or copy-number variation in cancer-related genes [81, 82]. Many of these mutations appeared to be induced by prolonged culture, since these changes were not observed in low passage cells. It has been proposed that such karyotypic aberrations occurred with adaptation to the original culture conditions used when the first few lines were being derived and expanded [83]. These observations emphasize the need for complete characterization of hESC lines, particularly the effects of long-term culture, and the design of guidelines for designating therapeutic-grade hESCs.

### C. Enrichment, Directed Differentiation, and Purification of hESC-Derived Cells

A primary safety concern when using pluripotent hESCs is their potential to form germ layer tumors. As discussed above, *in vivo* transplantation of undifferentiated hESCs in mouse models results in teratoma formation. Evidence of tumor formation has also been observed in differentiated hESC derivatives transplanted *in vivo* [84, 85]. Thus, it is essential that candidate hESC derivatives intended for use in cell transplantation are free of tumorigenic cells. Another concern is the differentiation of hESC-derived cells into unwanted cell types. For example, the engraftment of inappropriate muscle cells into damaged myocardium could alter the electrical activities of recipient tissue, provoking arrhythmias [86]. Thus, developing and further optimizing differentiation and purification protocols are necessary to minimize the generation of unwanted cell types for pre-clinical transplantation experiments and clinical therapy.

As discussed earlier, enrichment of specific cell types can be achieved using molecules introduced at critical time points during culture. However, many of these methods yield only moderate enrichment that is not yet scalable for clinical application. It may be desirable to enrich first for partially differentiated, proliferative hESC intermediates with specific cell fates. These could then be expanded before further differentiation into cells for therapy. For example, the expression of the cell surface antigen, CD133, on proliferating hESCs identifies cells predestined toward a neuroectodermal fate [26]. CD133-positive cells have been selected from cultures of undifferentiated hESCs, and have been observed to differentiate primarily into neuroectodermal cells *in vitro* and *in vivo* [26].

In the absence of specific cell surface antigens like CD133 to identify tissue-specific precursors, molecular beacons have been used to select for specific subpopulations of hESCs. King et al. [25] first demonstrated the utility of this system for isolating viable Oct4-expressing pluripotent hESCs in a specific and high-throughput manner. Molecular beacons are single-stranded oligonucleotides that generate fluorescent signals when bound to their target mRNAs, making these cells detectable and selectable by fluoresence-activated cell sorting. More importantly, molecular beacons have a short lifespan within cells and do not alter the function or genomic structure of hESCs. Thus, this method can be used to enrich for desired hESC-derived cell populations or used to select against unwanted cell types, such as undifferentiated hESCs that could form tumors [25].

### D. Circumventing Immune Rejection of Transplanted hESC-Derived Cells

Transplanted hESCs encounter immune rejection [87] because both proliferating and differentiated hESCs express class I and II HLA as well as minor histocompatibility antigens at levels sufficient to activate the immune system [87, 88]. Another potential barrier to hESC engraftment can occur through mismatch between hESC donor and recipient ABO blood group antigens [89-91]. While studies to determine the effects of ABO incompatibility on hESC transplantation are still lacking, this has long been a criterion for successful organ transplantation and thus, it is likely that ABO incompatibility between hESC-donor cells and the recipient would also trigger immune rejection.

Ideally, having genetically identical donor and patient cells is the best way to circumvent immune rejection. Thus, there is expressed interest in developing and using somatic cell nuclear transfer to generate patient-specific hESC lines. Using this technique, the DNA obtained from either a patient's skin or muscle cell would be transferred into an unfertilized egg that has had its DNA removed. Subsequently, the egg is artificially fertilized and allowed to develop until it reaches the blastocyst stage to derive hESCs. The resultant hESC line would have an immunologic profile matching the patient and could be used for cell therapy. This technique has been conducted successfully in animals using species-specific ESCs, but derivation of hESCs through somatic cell nuclear transfer has not yet been achieved.

Other strategies to generate hESC lines with the closest match to potential transplant patients include engineering “universal donor hESCs,” a blood antigen O cell in which the expression of HLA is suppressed, or chimeric hematopoietic cells derived from hESCs capable of inhibiting the immune response when co-transplanted with the desired hESC-derived cells [92]. Alternatively, creating banks of hESC lines representing HLA/ABO combinations that match the majority of potential patients has been proposed. Studies have provided estimates on how many hESC lines would be needed in order to support the needs of a specific population. Taylor et al. [93] estimated that approximately 150 hESC lines could provide an HLA match for most of the population in the United Kingdom. Alternatively, approximately 10 parthenote-derived hESC lines that are homozygous for HLA types could be sufficient for a majority of the population. Studies by Nakajima et al. [94] estimated that approximately 170 hESC lines, or 55 hESC lines with homozygous HLA types, would be sufficient for 80% of patients in the Japanese population. These findings demonstrate the feasibility of creating and maintaining a hESC bank with sufficient representation to support a large number of patients. However, in countries such as the United States, many more hESC lines would need to be established to serve its ethnically and genetically diverse population. Given the ethical issues and restrictions on hESC research, and the small number of approved hESC lines currently available, the creation of a hESC bank with a highly diverse collection of cell lines will undoubtedly face enormous challenges.

## V. Therapeutic Advantages of hESCs Over Other Stem Cell Sources

While not the focus of this review, other sources of human-derived stem cells are also being explored for use in clinical settings. Among these are adult stem cells and induced pluripotent stem cells (iPSCs). Unlike hESCs, these stem cells can be obtained directly from the individual to be treated. Thus, as a source of cells for therapy, they are able to circumvent the immunocompatibility issues that hamper many non-autologous transplantation therapies. Furthermore, the utilization of these stem cells in both clinical and basic research studies does not face ethical and political issues that otherwise surround the use of embryonic stem cells. However, adult stem cells and iPSCs have significant limitations as well that are potentially overcome by hESCs at this time.

Adult stem cells are derived from non-embryonic tissues and typically reside in their tissue of origin. Similar to hESCs, adult stem cells are capable of self-renewal. However, unlike hESCs, they have restricted potential and are able to differentiate only into cells from the tissue of origin. In some cases, adult stem cells are not able to generate all cell types of the tissue of origin, nor can they sustain growth over time. The latter problem has been encountered specifically in stem cells obtained from aging individuals [95, 96].

iPSCs are generated by reprogramming differentiated somatic cells into a pluripotent state. This can be achieved by inducing the expression of three core reprogramming factors Oct4, Sox2, and Nanog. Several methods have been employed to express these factors and induce pluripotency. Among these are retroviral, lentiviral, and adenoviral transduction, which carry the risk of permanent and harmful genomic integration. Indeed, some established iPSC lines are genetically unstable, exhibiting large-scale genomic rearrangements, copy number variations, and abnormal karyotype even in early passage stages [97, 98]. To minimize the possibility of mutagenesis caused by methods used to introduce the reprogramming factors, integration/plasmid-free strategies have been employed to express these factors and induce pluripotency such as synthetic RNA delivery, RNA virus transduction, or the addition of cell-penetrating purified recombinant proteins [99, 100]. However, these methods are significantly less efficient at generating reprogrammed pluripotent cells in comparison to viral integration. Furthermore, in some iPSC lines reprogrammed using non-integrating viral method, high levels of mutational changes were still observed [97].

In addition to genomic changes, recent studies have also revealed that iPSCs contain epigenetic features that indicate either incomplete or aberrant reprogramming. In particular, iPSC DNA methylation patterns are frequently reminiscent of the somatic cell of origin [101], suggesting that iPSCs are not completely reprogrammed into the naïve pluripotent state seen in hESCs. It is unclear whether the observed genetic instability and epigenetic imprinting accrued during reprograming or was present in the somatic cell of origin. Nevertheless, patient-specific iPSCs may be less suitable for the treatment of aging-associated diseases, since somatic cells from older individuals are more likely to contain genomic mutations and disadvantageous epigenetic programs. Thus, the safety and efficacy of therapeutic iPSCs as currently derived remain to be tested.

## VI. Conclusions

As cell replacement therapies are envisioned and realized, their use in the treatment of aging-associated diseases becomes a compelling prospect. hESCs provide much promise as a potential tool in designing such therapies, as well as in drug discovery. It is clear that there are still major scientific challenges as well as ethical and legislative issues that must be addressed. However, it is encouraging to see that clinical trials involving the use of hESCs have begun, and that extensive efforts are underway to efficiently, successfully, and safely differentiate hESCs into specific cell types. These studies will pave the way toward leveraging the therapeutic benefit of hESCs for regenerative medicine, particularly in aging-associated diseases.
